# Analysis of Damage Evolution in Concrete under Fatigue Loading by Acoustic Emission and Ultrasonic Testing

**DOI:** 10.3390/ma15010341

**Published:** 2022-01-04

**Authors:** Marc Thiele, Stephan Pirskawetz

**Affiliations:** Bundesanstalt für Materialforschung und-prüfung (BAM), 12205 Berlin, Germany; stephan.pirskawetz@bam.de

**Keywords:** concrete, fatigue, damage evolution, ultrasonic testing, acoustic emission

## Abstract

The fatigue process of concrete under compressive cyclic loading is still not completely explored. The corresponding damage processes within the material structure are especially not entirely investigated. The application of acoustic measurement methods enables a better insight into the processes of the fatigue in concrete. Normal strength concrete was investigated under compressive cyclic loading with regard to the fatigue process by using acoustic methods in combination with other nondestructive measurement methods. Acoustic emission and ultrasonic signal measurements were applied together with measurements of strains, elastic modulus, and static strength. It was possible to determine the anisotropic character of the fatigue damage caused by uniaxial loading based on the ultrasonic measurements. Furthermore, it was observed that the fatigue damage seems to consist not exclusively of load parallel oriented crack structures. Rather, crack structures perpendicular to the load as well as local compacting are likely components of the fatigue damage. Additionally, the ultrasonic velocity appears to be a good indicator for fatigue damage beside the elastic modulus. It can be concluded that acoustic methods allow an observation of the fatigue process in concrete and a better understanding, especially in combination with further measurement methods.

## 1. Introduction

Concrete is the most widely used structural material in the world for the construction of civil structures. In most applications, the fatigue behavior of concrete is not an issue. But for some specific applications, the fatigue behavior is of relevance, such as in bridges, offshore structures, or wind turbines. The fields of application for concrete are steadily expanding, with concrete structures becoming more and more filigree, and loads on structures with a cyclic character increasing. Therefore, the relevance of the subject of the fatigue of concrete will continue to increase.

In spite of the long history of research, there are still many gaps of knowledge with regard to the fatigue process of concrete. Reasons for this include the strong heterogeneous material structure, the resulting high scatter in material properties, the steady development of new concrete mixtures, as well as the expensive experiments that are necessary. Furthermore, the fatigue process in concrete is different under compressive and tension loading [1]. For laboratory investigations, the more localized fatigue damage under cyclic tension loading is principally easier to observe and to measure compared to the distributed fatigue damage under cyclic compressive loading. Extensive data and information are necessary to be able to explain the fatigue process in concrete in more detail. Caused by the complexity of the material structure as well as of the complex related fatigue process in concrete, this will be possible only by applying manifold destructive and nondestructive investigation methods. Destructive methods such as light microscopy, residual static strength, or scanning electron microscopy make it mostly possible to get an insight into a particular state of damage of the concrete structure, as applied in [1] and [2]. However, an observation of the development of these damages is not possible during the fatigue process due to the destruction of the sample. An attendant observation and investigation of the damage and their development is necessary for the investigation and explanation of the fatigue process in concrete. Nondestructive measurement methods are necessary to enable such investigation. Acoustic methods such as acoustic emission analysis and ultrasonic testing are very promising, nondestructive methods for the investigation of concrete. Both have already been used for measurements on metals and metal structure for a long time. However, for the investigation of concrete, the related experience and knowledge is more limited. Corresponding basic information for the acoustic emission can be found in [3]. For the application of acoustic emission and ultrasonic testing for the investigation of concrete, basic information can be found in [4]. It can be summarized that both methods were used in the past sporadically for the investigation of concrete but mostly under static loadings.

The acoustic emission enables the detection of acoustic events that were generated within the material. This method was used by [5] for the investigation of compressive low-cycle fatigue in concrete and in [6] for the investigation of compressive high-cycle fatigue. However, until now, the acoustic emission was mainly used for the investigation of static tests [7] and for cyclic bending tests on concrete with very localized crack areas [8,9]. Only in some cases the acoustic emission is used for the investigation of the fatigue behavior of concrete under cyclic compressive loadings, such as in [10], where the acoustic emission was used as a damage indicator as well as in [11]. In [2], the acoustic emission was used successfully for the specific investigation of the fatigue process in high strength concrete in combination with light microscopy, scanning electron microscopy, as well as strain measurements.

The ultrasonic testing enables the acquisition of integral changes in the material properties that are specifically influencing the propagation of elastic waves. Ultrasonic testing was used in [12] for cyclic compressive tests of concrete. It was used in recent years [13,14] for the determination of the dynamic elastic modulus of concrete in high-cycle compressive fatigue tests. Study [15] used the ultrasonic testing as a damage indicator for the comparison of static and cyclic compressive tests on concrete. However, this method was also used in cyclic tensile tests, such as found in [16] on the material level. Today, some embeddable ultrasonic sensors exist that can be implemented in concrete structures and also be used for acoustic emission, as presented in [17].

Moreover, there exist further developments such as coda wave interferometry, which is more sensitive to damage as the conventional ultrasonic testing. Basic principles are described in [18]. This method was applied on concrete components under static loading [19]. A transfer on fatigue loaded concrete is pending.

There are some already existing applications of acoustic emission and ultrasonic testing in the field of structural health monitoring with the objective to determine the current condition of concrete structures. A large demand exists, for example, in the sector of wind energy turbines, as stated in [20,21]. In this context, ref. [22] presents results from fatigue tests on concrete specimens under compressive loadings which were investigated with acoustic emission and ultrasonic testing. These tests were used as preparation for the application in monitoring systems at foundations of offshore wind turbines with the target to observe the condition of the structure, but not to investigate the damage process itself. In [23], an application of a reinforced concrete bridge deck is described with acoustic emission as well as ultrasonic testing for a monitoring of the state of the bridge deck.

**Figure 1:**
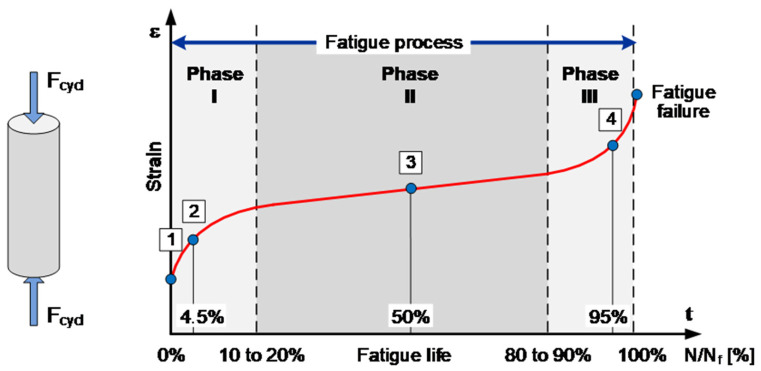
Schematic illustration of the characteristic development of the fatigue process in concrete and its three typical phases.

It is necessary to get a better understanding of the causal damage process in concrete under fatigue loadings (see Figure 1) to enable such applications in condition monitoring systems for cyclic loaded concrete structures. Only then will it be possible to identify suitable damage indicators which can be used for the evaluation of precise states of damage in concrete. Against that background, it can be stated that acoustic emission and ultrasonic testing were used so far only sporadically for the investigation of the damage process in concrete under cyclic compressive loadings. They were rarely used in combination usually with few sensors and only applied perpendicular to the loading direction. However, it is necessary to investigate the fatigue damage process as well as the measurability of fatigue damage to reach the goal of reliable monitoring systems for concrete structures for their condition evaluation.

The objective of the presented research was the investigation of the fatigue process (see Figure 1) and the related damage evolution in concrete by application of acoustic methods as well as the measurement of mechanical properties. The combination of these methods was applied for the investigation of the fatigue process of normal strength concrete under cyclic compressive loadings on the material level. The fatigue process was investigated under low-cycle (N_f_ < 10^3^ to 10^4^) and under high-cycle (N_f_ > 10^4^) [24,25] loadings to enable a better evaluation of the determined results. One aim was to verify the capability of the acoustic methods to detect the damage evolution in concrete based on this investigation. Furthermore, the intention is to get new conclusions about the causal fatigue damage processes in the material structure based on the additional combination with measurements of mechanical properties. Through the comparison of low-cycle and high-cycle tests, it will also be verified to what extent the fatigue process is different or similar in both cases. Based on all measurements and their comparisons, it is moreover possible to evaluate them with respect to their suitability as a damage indicator.

## 2. Materials and Methods

### 2.1. Material and Specimen

For the presented experimental investigation only one normal concrete mixture was used. It was the intention of the investigation to be focused on the general damage process in concrete under fatigue loading. Therefore, a normal strength concrete of grade C40/50 was used to achieve well-pronounced effects of the fatigue process on the material behavior. This allowed a better observation and measurement of the resulting damage evolution within the concrete specimen.

The grade of C40/50 is related to [26] DIN-EN-1992-1-1. Table 1 shows the composition of the concrete mixture.

**Figure 2:**
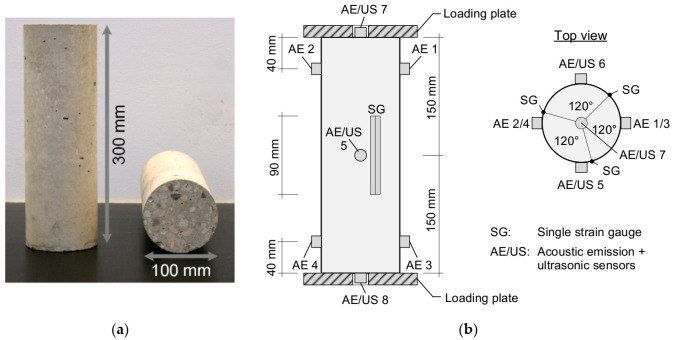
(**a**) Image of the tested cylindrical concrete specimen and its dimensions; (**b**) Illustration of the applied measurement arrangement of the concrete specimens for the fatigue tests.

Cylindrical specimens were produced based on this concrete composition with a ration of d/h = 1/3 (Figure 2a). This ratio was chosen to achieve a nearly undisturbed uniaxial state of stress at the middle section of the specimen height for the investigation of the fatigue process. Under consideration of the maximum aggregates with 16 mm, a diameter of 100 mm and a height of 300 mm were chosen for the specimen. Due to that knowledge, the ration of d/h = 1/3 and the precise geometry of 100/300 mm was used in several experimental investigations [27,28].

The specimens were demolded after one day and stored under water until the 7th day. Then the top and the bottom sides were grinded parallel before they were stored in a climate of 20 °C and 65% relative humidity until the date of testing. The testing of the specimen was done not before a minimum age of about eight months to avoid post-curing effects during the fatigue tests.

The related average of the 28-day compressive strength of the cylinders was about 55 N/mm^2^, and for the standard cubes of 150 mm an average compressive strength of 64 N/mm^2^ was determined.

### 2.2. Test Setup and Methods 

A servo hydraulic testing machine with a maximum load capacity of ±1 MN and a spherical calotte at the top loading plate was used for the fatigue tests. A cyclic compressive loading with constant amplitude was applied to the specimen with a frequency of 5 Hz. It was shown by several investigations in the past that the loading frequency influences the fatigue behavior of concrete [27,29,30]. However, the precise impact it has is still not sufficient investigated. The test frequency of 5 Hz was chosen to reach high numbers of load cycles within maintainable time periods.

**Figure 3:**
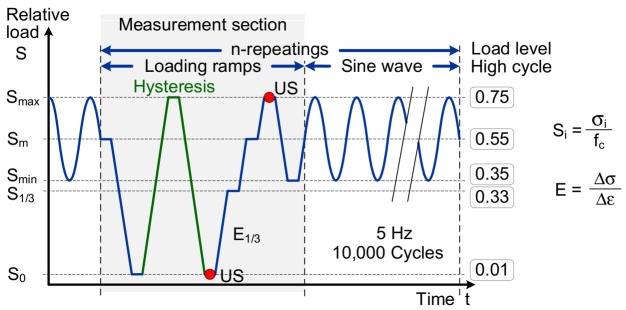
Schematic illustration of the applied loading sequence for the fatigue tests with intervals of static loading ramps for measurements within the cyclic fatigue loading.

The controller of the testing machine allows a complex programming of load functions, a synchronized acquisition of data from several sensors and can synchronize and control external measurement systems. This way it was possible to automatically interrupt the sinusoidal fatigue loading (interval with sine wave) in certain intervals for a sequence of monotonic loading ramps (measurement section). In this so-called measurement section, the data from the mechanical sensors were recorded (strains at S_0_ and S_max_, secant modulus between S_0_ and S_1/3_), the data acquisition of the acoustic emission system was enabled, and ultrasonic measurements were triggered at predefined load levels (at S_0_ and S_max_). The loading sequence is illustrated in Figure 3 and all relevant parameters are defined in Table 2.

It was planned to investigate the fatigue process for low-cycle (N_f_ < 10^3^ to 10^4^) and high-cycle (N_f_ > 10^4^) fatigue of concrete with the same loading and measurement concept. This allows the comparison of both fatigue processes and the identification of common and differing characteristics. Two different loading levels were chosen to achieve fatigue failure at about 10^6^ for high-cycle fatigue and at about 10^3^ to 10^4^ for low-cycle fatigue ([24] and [25]). All corresponding load values are given in Table 3 for the high-cycle fatigue tests (Series HCF). For the low-cycle fatigue tests (Series LCF) the corresponding values are given in Table 4.

The application of the sensors on the specimen for the nondestructive characterization of the fatigue process is illustrated in Figure 2b. As mechanical values, the longitudinal strains and applied force were measured. Three strain gauges with a length of 90 mm from TML type PL90 were placed in the middle of the specimen height in loading direction at each 120 ° of the circumference. The applied force was measured by the load cell of the testing machine. Strains and forces were measured quasi continuously during the measurement sections of the loading program (Figure 3). Based on the measurements of strain and force the elastic modulus was calculated additionally as secant modulus between load level S_0_ and S_1/3_.

Crack initiation, crack growth, and friction of crack surfaces in brittle materials are accompanied by the emission of transient elastic waves. These waves propagate through the material and can be detected and transformed into electrical signals by piezoelectric sensors on the surface. If such a signal crosses a predefined detection threshold, it is called a “hit” [31] and can be processed by an acoustic emission system and signal parameters such as the arrival time at the sensor, amplitude, energy, number of threshold crossings and duration, as well as the complete waveform, and be stored for analysis. A comprehensive introduction to acoustic emission testing including numerous examples for its application is given in [2].

For the detection of acoustic emission signals during the fatigue tests, an AMSY-5 Acoustic Emission system by Vallen Systeme GmbH was used. In total, eight piezoelectric sensors of type VS150-MS were acoustically coupled to the surface of the specimen by an adhesive putty as illustrated in Figure 2b. The VS150-MS sensors have a nominal frequency range from 100 kHz to 450 kHz with a peak frequency at 150 kHz. The bandpass filter of the AMSY-5 system was set to 50–300 kHz. In the low-cycle fatigue tests the data acquisition was enabled during the entire test. To reduce the amount of incoming data, it was necessary to set the detection threshold to 55 dB. In the high-cycle tests, the recording of acoustic emissions was activated only during the complete measurement section of the loading program (Figure 3). Additionally, due to the lower load level, the acoustic emission activity and hence the amount of incoming data are reduced. The detection threshold therefore could be lowered to 45 dB for the high-cycle tests. However, since only a qualitative assessment of the AE results of high- and low-cycle fatigue tests was done, this procedure is acceptable.

Triggered by the controller of the testing machine, electrical pulses can be sent individually to the VS150-MS sensors by the AMSY-5, turning the sensors into ultrasonic actuators and generating an ultrasonic pulse with a frequency of around 150 kHz. The ultrasonic velocity can then be calculated based on the time of flight of the ultrasonic pulse between different pairs of sensors. The arrival time of the ultrasonic wave at the receiving sensors was determined by the first threshold crossing of the signal.

The ultrasonic velocity was determined in parallel and perpendicular to the loading direction. For the measurement in parallel to the loading, two loading plates with holes for the sensors were used to attach the sensors directly at the top and bottom side of the specimen. For the perpendicular measurement, two opposite sensors in the middle height of the specimen were used. The measurement was performed at certain load levels in the loading program where the load was kept constant for the duration of the measurement. As shown in the loading program in Figure 3, the ultrasonic velocity was determined at the maximum load level (S_max_) as well as for the nearly unloaded condition (S_0_).

## 3. Results and Discussion

In this contribution only individual selected tests from the two performed test series (HCF series and LCF series) will be presented to allow a direct comparison of their results. However, these selected tests and their results exhibited typical and characteristic behavior and developments, which were observed within these both performed test series. A comparison will be done between low-cycle and high-cycle fatigue tests. Within the presented investigations, 14 fatigue tests were performed in total each for the low-cycle as well as for the high-cycle area.

### 3.1. Acoustic Emission

The acoustic emission measurement detects acoustic waves generated within the specimen. Sources of such acoustic waves can be mainly crack events such as crack initiation, crack growth. or the friction of crack surfaces. Therefore, acoustic emissions provide a good indication for the damage processes in concrete. They represent the damage growth as well as the current state of damage within the concrete. By using numerous sensors at different positions, it is also possible to localize the origin of single acoustic events. The localization algorithm is based on the arrival times of the signals from one source at different sensors which are strongly influenced by the acoustic velocity of the material which will be changing due to the proceeding material degradation caused by the fatigue loading. The analysis of acoustic emission signal parameters such as amplitude or average frequency can provide a deeper insight into the damage process, for instance, to identify tensile and shear or mixed mode cracking [32]. The focus within this paper is mainly on the acoustic emission activity, represented as the hit rate (number of acoustic emissions per time interval) in correlation to the changing stress in the specimen.

Acoustic emissions are determined by the microstructure of the material and the loading conditions. Results of acoustic emission measurement are therefore as individual as the microstructure of the specimen. They are strongly influenced by the location of the sensors in relation to the individual damage zones within the concrete specimen and also by the coupling condition of each sensor to the specimen. This increases the scatter in acoustic emission measurements additionally to the existing large scatter of the fatigue behavior of concrete. Therefore, it was not possible to consider absolute values from the measurements or to calculate average values from several tests. However, the characteristic of the result development is the same in all tests. This enables a qualitative consideration and assessment of these results. With this background only exemplary results from single tests, reflecting a typical behavior under certain loading conditions are presented.

**Figure 4:**
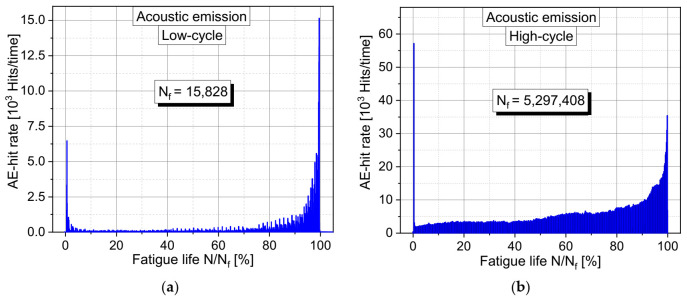
Similar characteristics in the development of measured acoustic emission activity over the relative fatigue life in comparison of (**a**) a low-cycle fatigue test with high stress level; (**b**) a high-cycle fatigue test with lower stress levels.

Some typical results of observed acoustic emission hit rates (detected signals per time interval) measured during the complete fatigue test are shown in Figure 4a for a low-cycle and in Figure 4b for a high-cycle fatigue test. These results are in good accordance with observations made in [5] or [9]. The three-phased development can be well-identified in the acoustic hit rate in both diagrams. Typically, the Phase I at the beginning in both cases is very shortly pronounced and extends only over the first loadings.

This Phase I exhibits a very high peak with many acoustic events during the first loadings. Subsequently, the acoustic hit rate passes over abruptly to a minimum of the hit rate. This peak is an indication of a short but very intensive crack activity. It can be assumed that during the first mechanical of the concrete structure, initial local stress concentrations in the material structure are reduced by microcracking and that redistributions of stresses are taking place. Existing microcracks out of the hydration process with a primary orientation perpendicular to the loading direction will likely be also activated and their crack surfaces be working into each other. This behavior is related to the so-called Kaiser effect. The acoustic activity is passing over abruptly to a minimum value after the stresses are more uniformly distributed in the material structure and existing cracks and crack surfaces are activated. This is the beginning of Phase II, which represents the largest part of the fatigue life and takes up to about 80% of the fatigue life. Here, a relatively stable state of the material structure seems to be reached with a slight continuous increase of the crack growth, or the formation of new cracks. After a certain moment, the acoustic activity increases significantly and passes over to an exponential increase. This identifies the transition to Phase III and indicates a significant increase in crack activities. The acoustic hit rate increases extremely up until the fatigue failure of the specimen and reaches similar or larger levels as observed in Phase I. It can be assumed that at this stage a grade of damage of the material structure is reached where in local areas in the material structure the bearing capacity is exceeded. This likely causes a density of microcracks that leads to a significant merge of microcracks to macrocracks that provoke an unstable crack growth in the specimen. Thereby a strong localization of damage and crack generation is activated. They finally lead to the fracture of the complete specimen due to macrocracks.

The process described above can be observed at the high-cycle as well as at the low-cycle fatigue tests in a very similar way. In low-cycle fatigue tests visible microcracks at the surface of the specimen can be observed at an earlier stage than in the high-cycle tests. This can likely be explained by the absolutely higher stress levels in the low-cycle tests. Therefore, it can be assumed that in both cases very similar damage processes with a comparable sequence in the total process are present.

A deeper insight into the dependency between mechanical loading and resulting acoustic activities within the material is possible by considering the measurements from specific sections of time taken from different stages of the fatigue process. In particular, the detailed measurements from certain measurement sections of the loading program (Figure 3) were analyzed. Here, the relation is investigated between the acoustic hit rate and stress sequence in the specimen.

**Figure 5:**
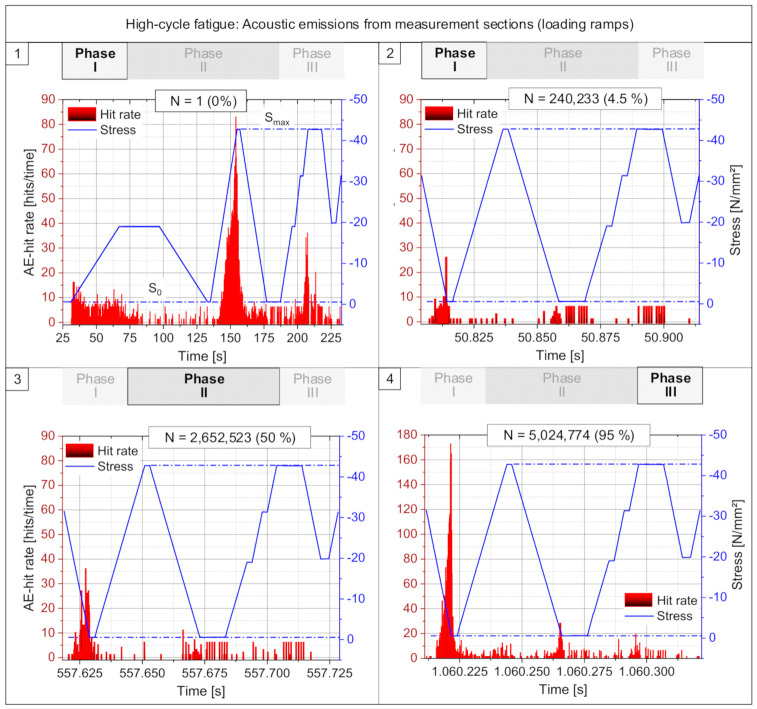
Comparison of acoustic emission activities at four different certain moments in lifetime of a high-cycle fatigue test taken from the measurement sections with static loading ramps.

Figure 5 shows four sections of the fatigue test with a duration of about 200 s (Diagram 1) and 110 s (Diagrams 2–4). Illustrated in Diagram 1 is the moment of first loadings at 0% fatigue life. Diagram 2 shows the time span directly after the abrupt decrease to a minimum of acoustic activity at about 4.5% of fatigue life in Phase I. The middle phase of the fatigue process in Phase II at about 50% is shown in Diagram 3 while in Diagram 4 the final part Phase III of the fatigue process is shown at about 95% of fatigue life. Significant differences can be seen in the characteristic of the relation between mechanical load and acoustic activity at the different phases of the fatigue process. The three phases of the fatigue process can be obviously differentiated in these results. It should be noted that the scaling of the acoustic hit rate is equal in Diagrams 1 and 3 while in Diagram 4, the scaling is extended since the values are higher in this final phase.

In Diagram 1 the first loading of the specimen is shown starting with a loading ramp for the determination of the elastic modulus and followed by a hysteresis as first loading up to the maximum load. In general, a very high level of acoustic hit rate is reached during the first loadings but mainly at the loading ramps and less at the unloading ramps. It can be seen very clearly at the first loading up to the maximum load, that the acoustic activity only starts to increase after the load level of the previous load ramp for the determination of the elastic modulus is exceeded. This behavior reflects the so-called Kaiser effect. At the following second loading to the maximum load the acoustic activity is significant smaller, but not zero.

Diagram 2 shows the moment at about 4.5% fatigue life where the acoustic activity has reached its minimum. At this moment acoustic emissions are still detected but at a very low level compared to the first loadings. Furthermore, the highest activities are now taking place mainly during the unloading phases. In contrast low values were observed in the loading phases. This change in behavior is an indication for a different damage mechanism and different processes between the phases are shown in Diagram 1 and 2.

This behavior is continued in principle in Phase II which is shown in Diagram 3. Here the highest acoustic activities can also be observed mainly at the unloading phases and less acoustic activities at the loading phases. This takes place on a slightly increased level and represents a stable process in Phase II. The highest acoustic activities are here observed at the first unloading after leaving the sinus loading section. This sinus loading is running on a relative high stress level (S_m_) compared to the nearly total unloading (S_0_) in the measurement section. Obviously, stress states and creep deformations (visco-elastic and visco-plastic deformations) were generated in the material structure during the sinus loading phase which was running on an average load level at S_m_ (see Figure 3). Likely, a significant relaxation of the concrete structure is taking place in case of the following nearly total unloading. This relaxation likely causes movements and deformations at the existing crack structure as well as at a crack growth or crack initiation. A result of that would be an increased crack activity which fades at repeated unloading phases.

Finally, it can be seen in Diagram 4 that the acoustic activities are increasing significantly and mainly at the first unloading. It could be assumed that in Phase III the existing crack state is now at such a large extent that due to the relaxation at the first unloading, a very high acoustic activity is released. This is indicative of the unstable damage and crack state at the material structure which now leads to an accelerated increase of the acoustic activity. A state of cracks and damage is reached at the end of Phase III which makes it no longer possible for the concrete specimen to sustain the applied cyclic load, and the fracture of the specimen takes place.

### 3.2. Ultrasonic Testing

The ultrasonic velocity in isotropic elastic materials in general is determined by its elastic constants. Damage at the structural level alters both the macroscopically measurable material properties and the acoustic properties. Generated microcracks or bulking of the material structure lead to a reduction of the ultrasonic velocity.

Within the presented investigations the measurement of the ultrasonic velocity was performed perpendicular as well as in parallel to the loading direction (see also Figure 2b). Due to the applied uniaxial cyclic loading, an anisotropic state of damage can be expected.

**Figure 6:**
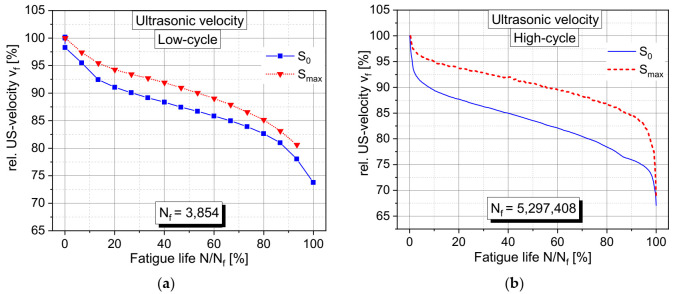
Comparison of the developments of relative changes in ultrasonic velocity during the fatigue life measured at maximum stress (S_max_) and at nearly unloaded conditions (S_0_) for (**a**) a low-cycle fatigue test with high stress level; (**b**) a high-cycle fatigue test with lower stress levels. Both tests measured in horizontal direction at the middle of the height of the specimens.

In Figure 6, a comparison is shown of the relative development of the ultrasonic velocity measured in the middle of the height of the specimen in a horizontal direction (perpendicular to the loading) for a low-cycle and a high-cycle fatigue test. The ultrasonic velocity exhibits a very similar behavior in both cases which is an indication as well for a very similar damage process. A slightly larger reduction in ultrasonic velocity is observed at the high-cycle test. However, this could also be a consequence of the significantly higher number of measurements during the high-cycle tests compared to the low-cycle tests. The ultrasonic measurement took place only at the measurement section of the loading program as illustrated in Figure 3. Therefore, the acquisition of the ultrasonic velocity is more influenced by a lower number of measurement points at the low-cycle fatigue tests, especially at the end of the fatigue test.

Very clearly pronounced is the three-phased development of the ultrasonic velocity. However, here the extent of Phase I is larger compared to the developments of the acoustic activities. Here, a stable and continuous change during Phase II in the ultrasonic velocity also takes place but with a stronger changing rate compared to the acoustic activity. This change in Phase II indicates a permanent increase in damage during this phase which is well-detectable with the ultrasonic velocity. This observation is in good accordance with the observations made by [13,15].

Significant differences can be observed between the development of the ultrasonic velocity at the fully loaded state (S_max_) and at the nearly unloaded state (S_0_). This applies to the low-cycle as well as to the high-cycle tests. The impact of the fatigue damage on the reduction of the ultrasonic velocity is larger at the unloaded condition compared to the loaded condition. This means that the concept of mainly load parallel oriented fatigue cracks, which are opened under loading conditions, cannot explain this observed load-dependent behavior. The state of the fatigue damage is obviously much more complex in the concrete structure [1].

**Figure 7:**
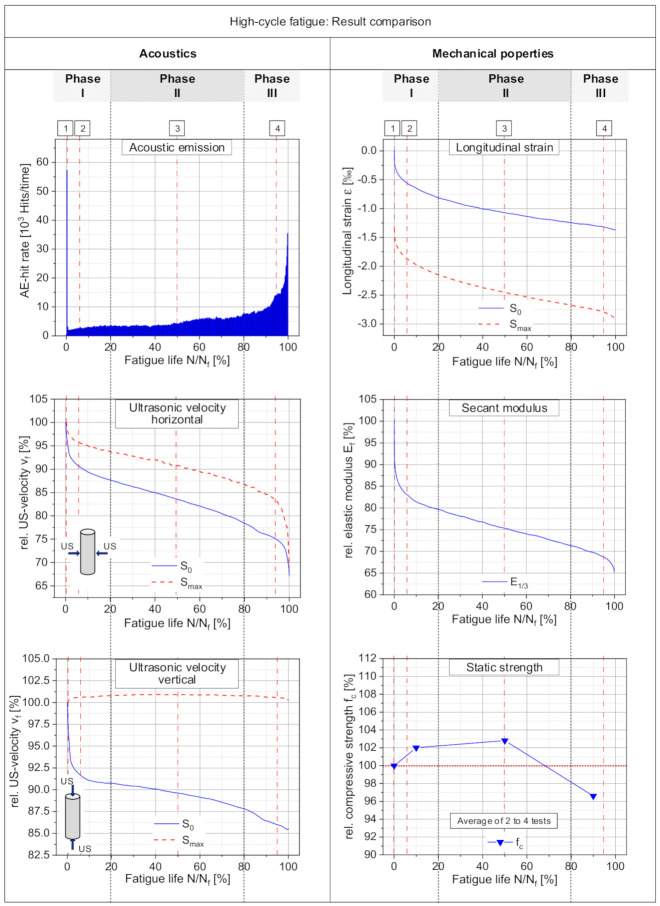
Comparison of results from acoustic measurements of the fatigue process with three mechanical properties. All results taken from one fatigue test, except the static strength.

Additionally, in the diagram at the bottom left of Figure 7, the result of the vertical (load parallel) measurement of the ultrasonic velocity of the same test is shown. Here, a clear difference can be seen between the behavior of the perpendicular and parallel measurements, especially under loading conditions. This clearly indicates the anisotropy of the fatigue damage caused by an uniaxial loading. The difference between the loaded and unloaded conditions is much largely pronounced at the load parallel measurement of the ultrasonic velocity. Here, both curves exhibit a three-phased development, as well. However, under loading conditions, no reductions, but rather a slight increase, of the ultrasonic velocity can be observed. Only at the end of Phase III a slight decrease can be seen. However, for the unloaded condition, a very similar development as observed in the horizontal measurements takes place. A significant decrease can be seen which is not so strongly pronounced as observed at the horizontal measurements. This observed behavior is likely an indication for damage and cracks, which are oriented mainly perpendicular to the loading direction. They are pressed together under load and opened at unloaded conditions.

### 3.3. Acoustics vs. Mechanical Properties

A comparison is done with the measurements of mechanical properties to enable an assessment of the acoustic measurements of the fatigue process of the high-cycle tests. This comparison is focused on the high-cycle fatigue tests since this is more relevant for the practical application of concrete and a completely identical set of data was unavailable for the low-cycle fatigue tests. All measured acoustic signals are influenced by mechanical properties and the damage of the material. Therefore, appropriate correlations are expected with the development of mechanical properties.

To illustrate such correlations, a comparison is given in Figure 7 of the acoustic activity, the ultrasonic velocity perpendicular and vertical to the load with the development of the mechanical properties for longitudinal strains, and the elastic secant modulus and residual static strength of the specimen. This will give a more complete image of the effects of the damage process caused by fatigue loading. Based on this, conclusions can be drawn on the causal fatigue process itself.

In Figure 7, individual results are compared of one high-cycle fatigue test with a number of cycles to failure of 5,297,408. Only the results of the residual static strength are taken from several different tests. For this purpose, individual specimens were tested with a specific detection method until they reached certain states of the fatigue life. They were then tested under static loading to determine the residual strength. The shown acoustical and mechanical measurements exhibit in principle similar and three-phased developments. However, the single phases are partially pronounced differently and have varying lengths. This is, of course, a result of the fact that the fatigue damage influences the single measurement values to a different extent and furthermore an anisotropic state of damage is generated. Moreover, not only does a reduction of values takes place, but increases were also observed in the acoustic and mechanical measurements. This happens at the load parallel ultrasonic velocity under loading condition (S_max_), as well as for the development of the residual static strength (Figure 7 left and right column bottom line).

In general, a very good correlation can be observed between acoustic and mechanical measurements. The load parallel ultrasonic velocity under loading condition (S_max_) obviously correlates with the development of the residual static strength, as mentioned before. The ultrasonic velocity perpendicular to the loading correlates well with the development of the strains as well as with the elastic secant modulus (Figure 7, left column middle and right column, top and middle). The ultrasonic velocity also gives an indication to the anisotropic damage caused by the uniaxial fatigue loading. The acoustic emissions allow conclusions on the state of cracks as well as on the growth of cracks within the material structure caused by fatigue loading. Part of the fatigue process are obviously cracks or damage with a horizontal orientation and local compaction in the material structure. They are active only under unloaded states, as seen in the load parallel ultrasonic measurement. However, they can contribute to the generation of acoustic signals as well as to the decrease of ultrasonic measurements perpendicular to the load. Under loaded conditions, they are closed and compacted. Therefore, the load parallel ultrasonic measurement is approximately constant during the test. Furthermore, the static strength is not reduced by these damages and can be increased by effects of creep and compaction in the concrete structure. In contrast, the ultrasonic measurement perpendicular to the load is more effected by phenomena which are active in the horizontal direction. The longitudinal strain more correlates with the ultrasonic measurement perpendicular to the load, since this strain is coupled to the resulting perpendicular strain and to damages which are active in the horizontal direction. Damage or crack states which reduce the residual static strength seem to be generated only at the last Phase III of the fatigue process.

Finally, each measurement allows specific conclusions related to the fatigue process. Therefore, based on all the performed measurements, it is possible to derive more and better conclusions about the fatigue process at the material structure level of concrete.

## 4. Conclusions

Measurements of mechanical properties, acoustic emission activity and ultrasonic velocity were combined to study the evolution of damage in normal strength concrete in high-cycle and in low-cycle compressive fatigue tests. Conventional cyclic load functions were automatically interrupted by measurement phases—sequences of linear load ramps and dwell times. This way, it was possible to determine mechanical and acoustic characteristics on certain load levels and to correlate these characteristics to the fatigue life. It can be summarized that all tested measurement methods are suitable for characterizing the damage evaluation and in distinguishing the three phases of fatigue life. The change in material properties until the final failure remains in the range of the scatter of material properties of different batches. Without information about initial material properties, it is therefore not possible to identify the stage of damage through single measurements of the ultrasonic velocity or the secant elastic modulus. Based on the change in material characteristics, repeated measurements during fatigue load could allow an estimate of which phase of damage is reached. A deeper analysis of combined acoustic emission parameters or a signal-based analysis including pattern recognition can help to identify the stage of damage in more details.

In this study on normal strength concrete, the damage evolution in high- and low-cycle fatigue tests is very similar. Future studies under similar conditions could likely be focused therefore on low-cycle fatigue tests.

In general, all measurement methods described can be applied to real structures. Since the range of the sensors is limited to a few meters at most, the monitoring of the structure should be concentrated on the most vulnerable points. Even if the current stage of damage cannot be identified clearly, the change rate of measurement results can warn of structural failure.

## Figures and Tables

**Table 1:** Composition of the normal strength concrete used for the fatigue tests.

w/c Ratio	Water	Cement	Super Plast.	Aggregates [kg/m^3^]		
				Grading		
[–]	[kg/m^3^]	[kg/m^3^]	[kg/m^3^]	0/2	2/8	8/16
0.461	169	375	5.25	544	581	690

**Table 2:** Relevant parameters for the fatigue tests.

Parameter	Description	Formular
ε	Strain (longitudinal)	-
σ	Stress (longitudinal)	-
f_c_	Static compressive strength	-
S	Relative stress level	S = σ/ f_c_
S_1/3_	Relative stress level	S_1/3_ = 1/3 ˖ f_c_/f_c_
E	Secant elastic modulus	E = Δσ/Δε
E_1/3_	Secant elastic modulus	$E_{1/3}=\frac{\sigma \;\left(S_{1/3}\right)-\sigma \;\left(S_{0}\right)}{\varepsilon \;\left(S_{1/3}\right)-\varepsilon \;\left(S_{0}\right)}$
N	Number of load cycles	-
N_f_	Number of load cycles at failure	-

**Table 3:** Series HCF—Load and stress values for the cyclic loading of the high-cycle fatigue tests.

Load	S_0_	S_1/3_	S_min_	S_m_	S_max_
S [–]	0.01	0.33	0.35	0.55	0.75
F [kN]	5	145	152	239	326
σ [N/mm^2^]	0.66	19.0	19.9	31.3	42.7

**Table 4:** Series LCF—Load and stress values for the cyclic loading of the low-cycle fatigue tests.

Load	S_0_	S_1/3_	S_min_	S_m_	S_max_
S [–]	0.01	0.33	0.425	0.625	0.825
F [kN]	5	145	185	272	360
σ [N/mm^2^]	0.66	19.0	24.2	35.6	47.2

## Data Availability

The data presented in this study are available on request from the corresponding author. The data are not publicly available due to privacy reasons.
